# Appendicitis Band Syndrome: A Case Report and Literature Review

**DOI:** 10.7759/cureus.94858

**Published:** 2025-10-18

**Authors:** Amina Babana El Alaoui, Salaheddine Abdennebi, Youssef Ouazzani Touhami, Abdelkader Belkouchi, Omar Belkouchi

**Affiliations:** 1 General Surgery, Ibn Sina Hospital, Faculty of Medicine and Pharmacy, Mohammed V University, Rabat, MAR; 2 Surgery, Faculty of Medicine and Pharmacy, Mohammed V University, Rabat, MAR; 3 Surgery, Moulay Abdellah Hospital, Salé, MAR

**Keywords:** acute appendicitis, appendiceal tie, appendicitis band syndrome, bowel strangulation, small bowel obstruction, surgical emergency

## Abstract

Appendicitis band syndrome (ABS) is difficult to diagnose preoperatively due to nonspecific clinical and radiological findings. Most cases are identified during laparotomy performed for presumed small bowel obstruction. Management ranges from simple appendectomy to extensive bowel resection, depending on the viability of the involved segments. Imaging, particularly CT, may show indirect signs such as transition points, but confirmation often requires surgical exploration. We present the case of a 27-year-old woman with no prior medical history, who presented with a two-day obstipation. The CT scan showed bowel obstruction secondary to a terminal ileum stenosis. We performed a laparoscopic exploration during which we found a portion of the small bowel had herniated through a loop formed by an inflamed appendix, and a laparoscopic appendicectomy was successfully performed. The patient showed no complications in the two-week and one-month follow-ups. Given its diagnostic difficulty and potentially severe complications, ABS should be considered in all cases of small bowel obstruction of unknown origin, particularly in patients with no history of prior abdominal surgery. Prompt surgical intervention is critical to reduce morbidity and mortality. Tailoring the surgical approach, laparoscopic or open, based on clinical context, remains essential for optimal outcomes.

## Introduction

Intestinal obstruction remains a common and potentially life-threatening surgical emergency, with a wide range of underlying causes. While postoperative adhesions, hernias, and tumors account for the majority of cases, rare etiologies must be considered, particularly when classical causes are not evident. One such rare and underrecognized cause is intestinal obstruction secondary to acute appendicitis, with only a few cases documented in the literature, reported in adults as well as neonates [1].

While small bowel obstruction has numerous aetiologies, acute appendicitis is an uncommon cause. Appendicitis band syndrome (ABS) is a unique pathological condition wherein an inflamed and elongated appendix forms a constricting loop by adhering to adjacent anatomical structures such as the cecum, small intestine, or mesentery, leading to mechanical bowel obstruction and, in severe cases, strangulation and ischemia. Remarkably, this condition can arise in the absence of prior abdominal surgery, thus distinguishing it from adhesion-related obstructions. The highly mobile nature of the appendix, combined with its anatomical variability and susceptibility to inflammation, contributes significantly to the formation of these constrictive bands, especially when the appendix is elongated [2].

The true nature of the pathology is typically discovered only during surgical exploration. Imaging, especially computed tomography (CT), may suggest bowel obstruction or a right iliac fossa mass, but definitive identification of an appendiceal band is rarely achieved preoperatively. Preoperative diagnosis remains highly challenging, as the condition is typically identified only during laparotomy. Management may range from simple appendectomy to more extensive procedures such as bowel resection or right colectomy, depending on the intraoperative findings [3].

## Case presentation

We report the case of a single childless 27-year-old woman, with no medical history, no previous abdominal or pelvic surgery, or any comorbidity, who presented to the emergency department with severe abdominal pain, a two-day obstipation, vomiting, and apyrexia.

On physical examination, there was pelvic tenderness, while the remaining abdominal quadrants were non-tender. The abdomen was distended, and the patient was tachycardic with a heart rate of 101 beats per minute.

The patient underwent blood tests, the results of which are shown in Table 1.

**Table 1:** Patient Key Blood Test Parameters

Test	Value	Unit
Hemoglobin	11.7	g/dl
White Blood Cells (WBC)	8.900	/mm³
Platelets	350 000	/mm³
C-Reactive Protein (CRP)	2.4	mg/L
Sodium	140	mEq/L
Potassium	4.1	mEq/L
Urea	0.4	g/L
Creatinine	9	Mg/L

Several causes of intestinal obstruction were considered during clinical investigations, including, but not limited to, a congenital adhesion, cecal volvulus, and other neoplastic etiologies. A gynecological cause could not be ruled out either.

**Figure 1:**
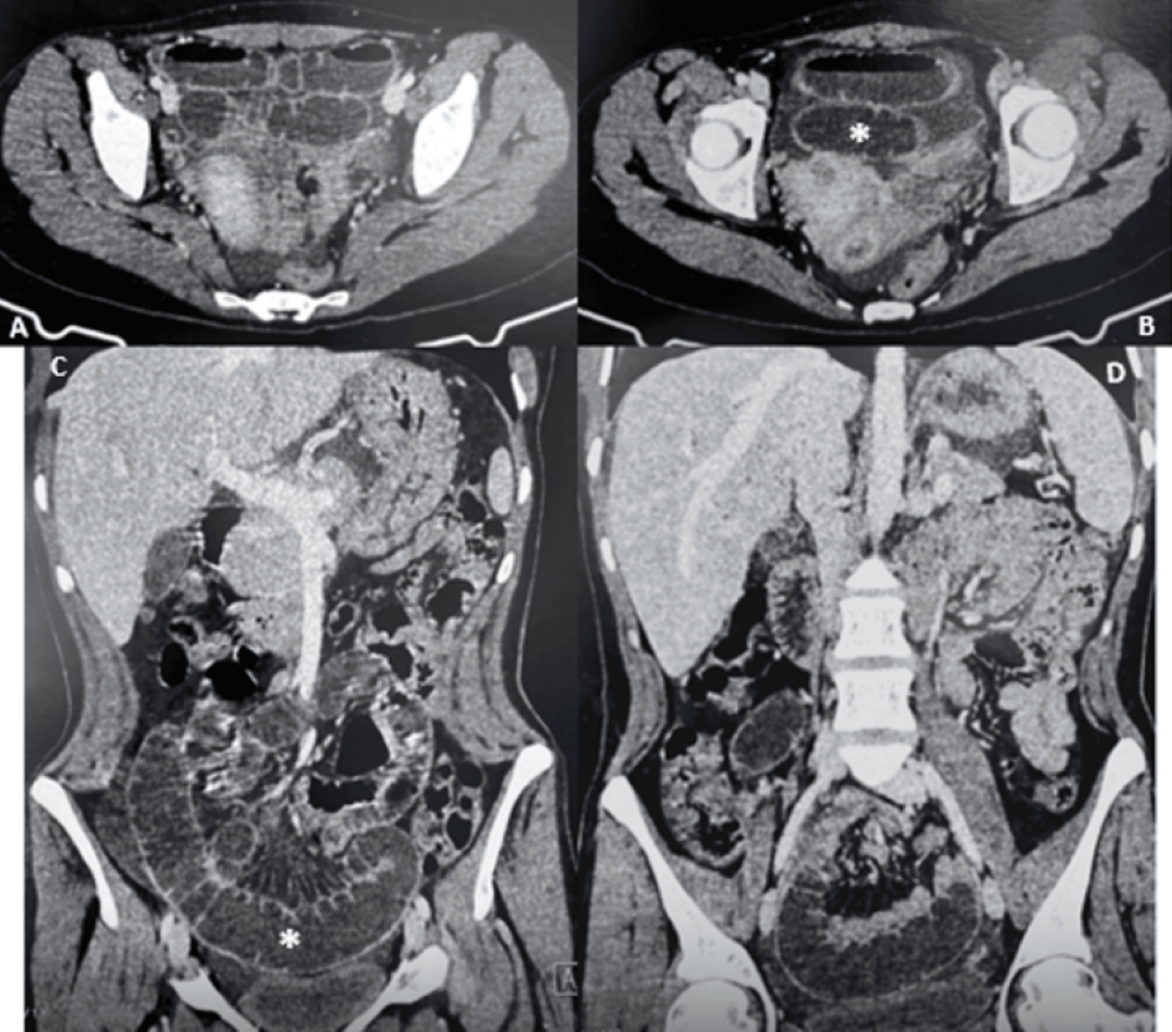
CT scan showing acute bowel obstruction, dilated ileum and air-fluid levels (*). (A) and (B) Axial view and (C) and (D) Coronal view

The CT scan revealed a bowel obstruction secondary to a stenosis of the terminal ileum, associated with peritoneal fluid accumulation (Figure 1).

**Figure 2:**
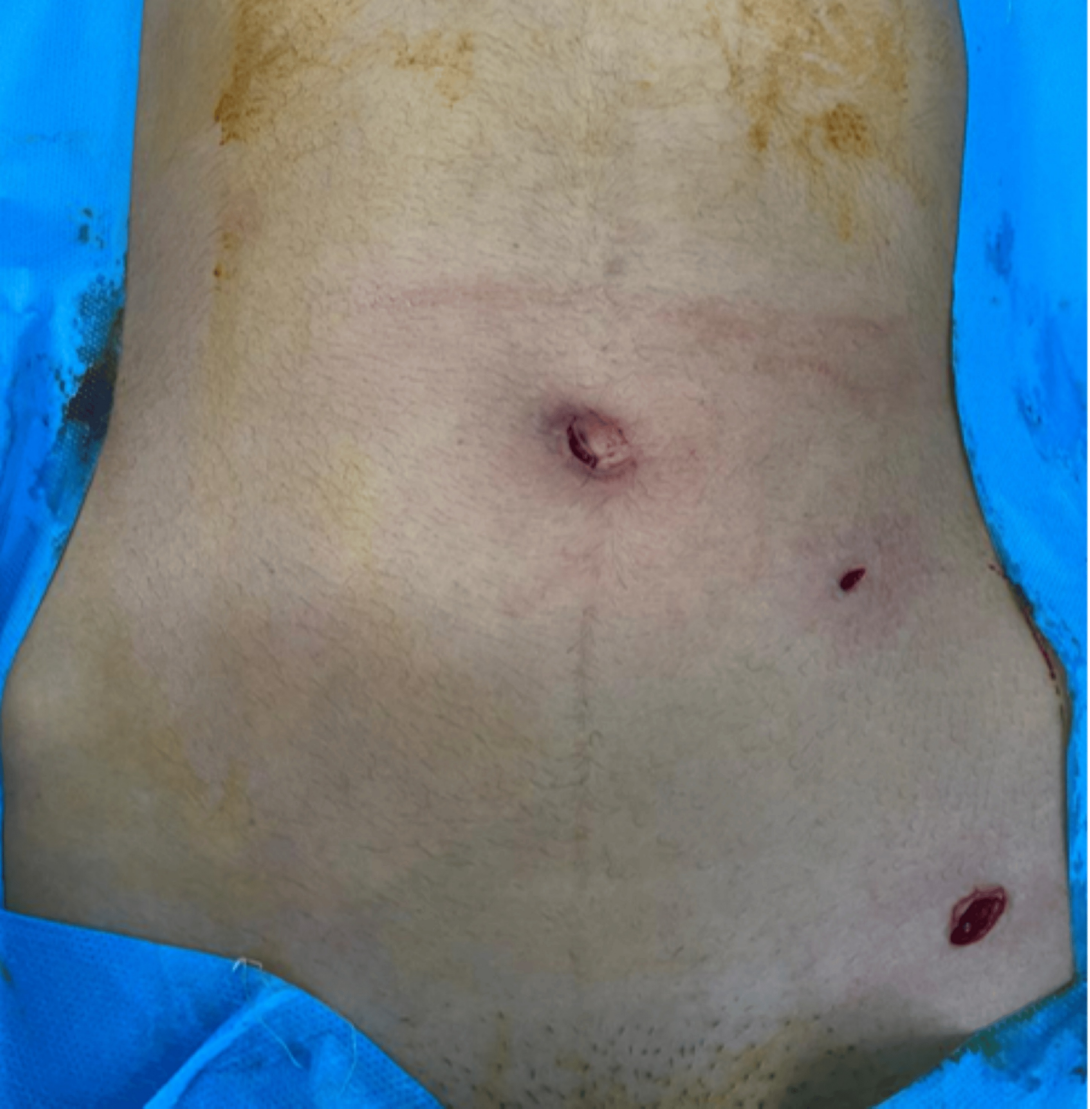
Preoperative image showing trocar placements

**Figure 3:**
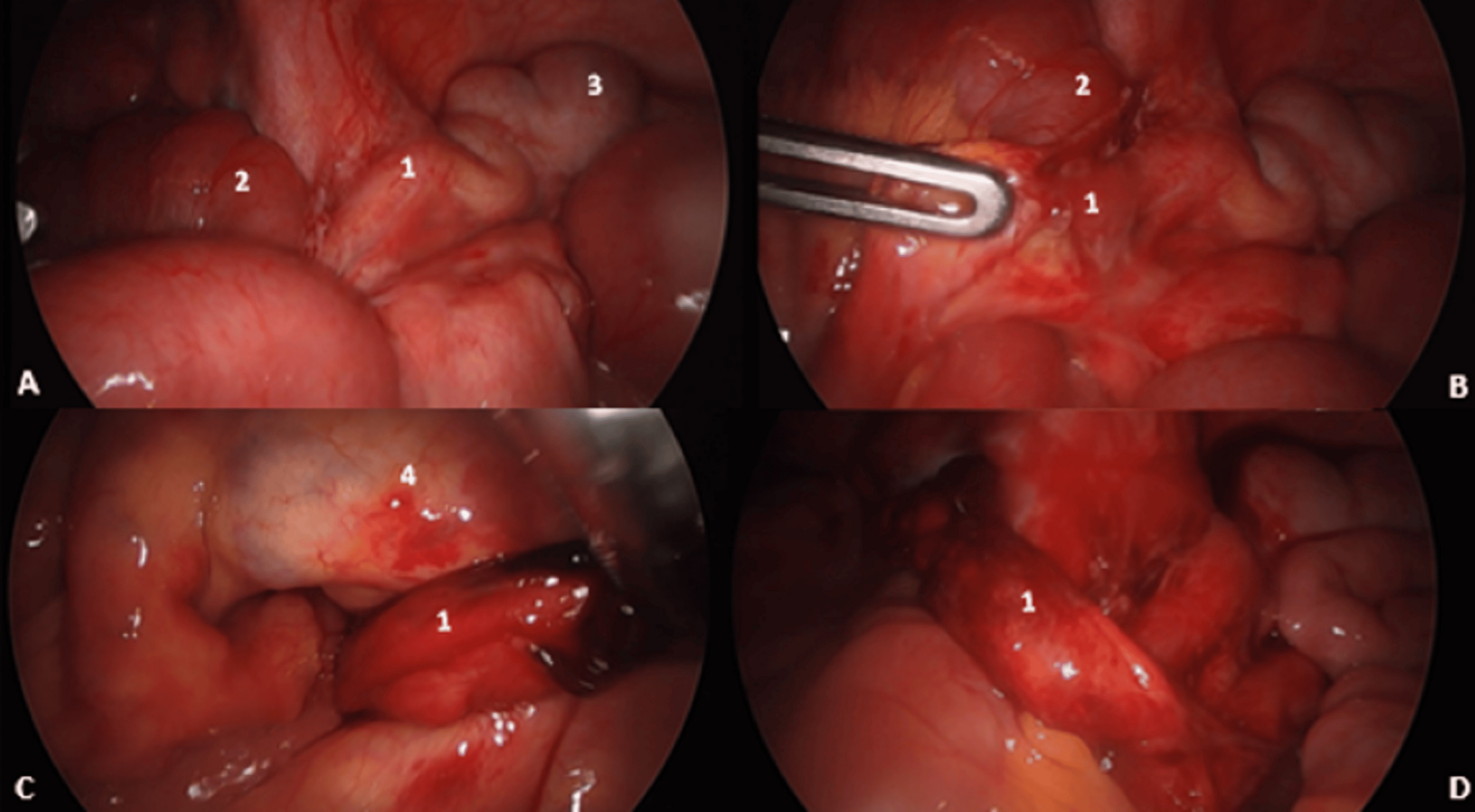
Preoperative view showing (A) appendix at the exploration, (B) appendix held by a grasper, adherent to the ileum, (C) tip of the appendix adherent to the meso-ileum, and (D) length of the appendix after freeing the ileum loop (1) Appendix, (2) ileum, (3) cecum, and (4) meso-ileum

**Figure 4:**
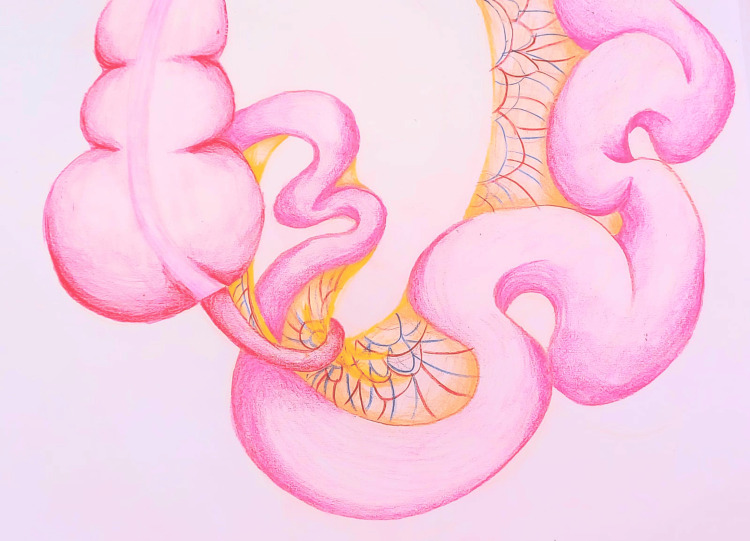
Description of the inflamed appendix encircling the ileum and its mesentery, associated with bowel distension Original drawing made by the article's author (Babana El Alaoui Amina)

We performed a laparoscopic exploration under three ports (Figure 2). Upon exploration, we found some pelvic fluid. The ovaries and uterus showed no abnormalities. Through the ileocecal region, we found a dilated small bowel, as it had herniated through a loop formed by an inflamed long appendix (Figure 3). The tip of the appendix was herniated below the meso-ileum (Figure 4), which was dissected and freed, and then the remaining body of the inflamed appendix was detached from the anterior wall of the ileum, without any injury.

**Figure 5:**
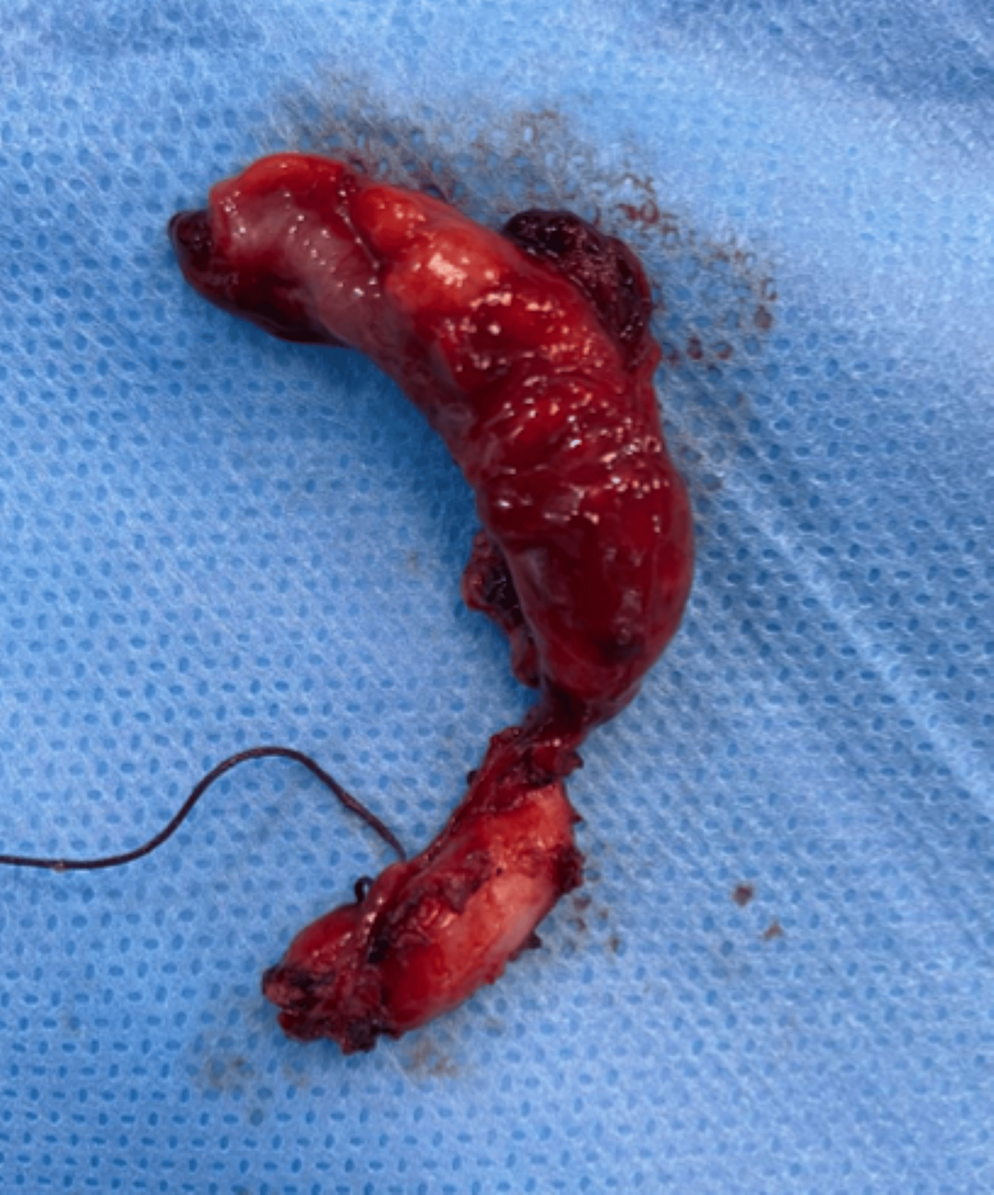
Image showing the surgical specimen (removed appendix)

As the herniated bowel was healthy, no bowel resection was needed. The base was closed using a knot ligature, and the appendix was resected. Thus, appendectomy was completed laparoscopically (Figure 5).

The patient was discharged on postoperative day two and showed no complications in the two-week and one-month follow-ups.

## Discussion

In 2009, Bhandari and al classified intestinal obstruction caused by appendicitis into four types: adynamic or paralytic ileus, mechanical bowel compression, bowel obstruction due to strangulation, and caused by mesenteric ischemia [2]. Adynamic obstruction, or paralytic ileus, is the most common form of obstruction associated with appendicitis, occurring in 1-5% of cases. In contrast, ABS is a rare entity, with only a few cases reported in the literature [2,4].

ABS is characterized as a mechanical obstruction and strangulation caused by an inflamed appendix encircling and adhering to the caecum, colon, small intestine, or posterior peritoneum. It can occur even in patients with no history of prior surgery [2,4]. ABS has been reported across all age groups, including neonates and children [5-7].

The appendix is a mobile organ with many variations in its position and length. Acute appendiceal inflammation is believed to initiate the formation of an appendiceal band around bowel loops or adjacent structures. As the inflammatory process progresses, the appendix may adhere to surrounding tissues such as the cecum, retroperitoneum, ileal mesentery, or the ileum itself, creating a defect through which part of the bowel herniates. This entrapment results not only in mechanical intestinal obstruction but also in ischemia of the bowel and appendix due to compressive forces. An increased length of the appendix seems to facilitate these phenomena [2,4,5].

ABS or appendiceal-tie syndrome has two main aetiologies: either the tip of the appendix attaches to the mesentery surrounding the ileal loop, causing compression of the ileal lumen, or adheres to the intestinal serosa, leading to both luminal compression and torsion of the bowel loop [4,5,8].

Complications are not only intestinal obstruction and strangulation of the entrapped bowel, but also the ischemia of the appendix itself due to compression or gangrene, all of which can lead to a fecal peritonitis [1,7].

Patients may present with predominant features of appendicitis accompanied by signs of bowel obstruction, or conversely, with bowel obstruction symptoms that obscure the underlying appendiceal cause. In many cases, appendicitis is only identified intraoperatively as the source of obstruction. Therefore, appendicitis should be considered a potential cause in cases of mechanical bowel obstruction of unknown origin, particularly in elderly patients [5].

Preoperative diagnosis is very challenging; however, the cardinal symptoms remain abdominal pain, vomiting and abdominal distension [7].

In the early inflammation phase, CT may assist in diagnosis by revealing a transition point, features of bowel obstruction, a mass in the right iliac fossa, or an appendicular band (or tourniquet) at the transition point. Nonetheless, definitive diagnosis is most often established during surgery [6,7].

Since preoperative diagnosis is challenging, early operative management is a crucial step to improve postoperative prognosis, as delays in surgery significantly increase the risk of fatal outcomes [4].

Although laparoscopic surgery offers several advantages, the choice between laparoscopy and open surgery in cases of ABS should be tailored to each case. Key considerations include the patient’s clinical status (such as hemodynamic instability or underlying comorbidities, any prior abdominal or pelvic surgery ...), the complexity of the case (including severe inflammation, abscess formation, perforation, peritonitis, or extremely dilated bowel loops), and the surgeon’s expertise. These factors may impact the selection of the appropriate surgical approach [6].

Management of ABS depends on the extent of strangulation and the segment of bowel involved. Surgical exploration is performed to identify any other cause of obstruction. Surgical intervention may vary from a simple appendectomy to a right colectomy, depending on intraoperative findings [7].

When diagnosed early, and the herniated bowel remains viable, simple release of the appendiceal band followed by appendectomy is typically sufficient, as demonstrated in our case. However, if the bowel is nonviable, resection with either primary anastomosis or stoma might be necessary [7].

## Conclusions

ABS although rare, represents an underdiagnosed cause of mechanical small bowel obstruction. Its clinical presentation is very variable, ranging from classic appendicitis to predominant features of intestinal obstruction, frequently masking the underlying pathology until surgical exploration is performed. The formation of a constricting appendiceal band due to inflammation, fibrosis, and anatomical variability highlights the appendix's potential for complex surgical pathology.

Imaging, particularly CT, lacks definitive specificity. Early surgical intervention is fundamental for reducing morbidity and mortality. The surgical approach, laparoscopic or open, should be carefully tailored to each patient. Prompt recognition and pre-emptive surgery are essential in improving outcomes for this entity.
